# Reduced Lattice Thermal Conductivity in Thermoelectric α‑MgAgSb via Sb_2_Te_3_ Powder Atomic Layer Deposition

**DOI:** 10.1021/acsami.5c23388

**Published:** 2026-02-06

**Authors:** Irene García Santamaría, Amin Bahrami, Angelika Wrzesińska-Lashkova, Jaroslav Charvot, Andrei Sotnikov, Lars Giebeler, Yana Vaynzof, Filip Bureš, Pingjun Ying, Kornelius Nielsch

**Affiliations:** † 28394 Leibniz Institute for Solid State and Materials Research Dresden, Dresden 01069, Germany; ‡ Institute of Materials Science, Technische Universität Dresden, Dresden 01062, Germany; § Chair for Emerging Electronic Technologies, Technische Universität Dresden, Dresden 01187, Germany; ∥ Institute of Organic Chemistry and Technology, Faculty of Chemical Technology, 495954 University of Pardubice, Pardubice 53210, Czech Republic; ⊥ Institute of Applied Physics, Technische Universität Dresden, Dresden 01062, Germany

**Keywords:** thermoelectric, energy harvesting, α-MgAgSb, powder atomic layer deposition, Sb_2_Te_3_

## Abstract

α-MgAgSb is an environmentally friendly alternative to traditional tellurium-based thermoelectric materials for near room temperature applications. In this study, we enhance the thermoelectric properties of α-MgAgSb by introducing a secondary Sb_2_Te_3_ phase using powder atomic layer deposition (powder ALD), with the aim to modify phonon scattering mechanisms and reduce the lattice thermal conductivity. Powder ALD is a thin-film deposition technique that allows for the deposition of self-limiting monolayers on high aspect ratio surfaces, enabling the conformal coating of nanopowder regardless of particle morphology. Sb_2_Te_3_ was selected as the coating material due to its oxygen-free synthesis route and its potential for good interfacial compatibility with the α-MgAgSb powders. Our results reveal a 10% decrease in lattice thermal conductivity of bulk α-MgAgSb as the powder ALD coating thickness increases from pristine to 20 cycles of Sb_2_Te_3_, without affecting the primary phase purity. Our findings highlight the effectiveness of nonoxide powder ALD coatings in suppressing lattice thermal transport, offering a promising pathway for interface-engineered, low-toxicity thermoelectric materials.

## Graphical abstract

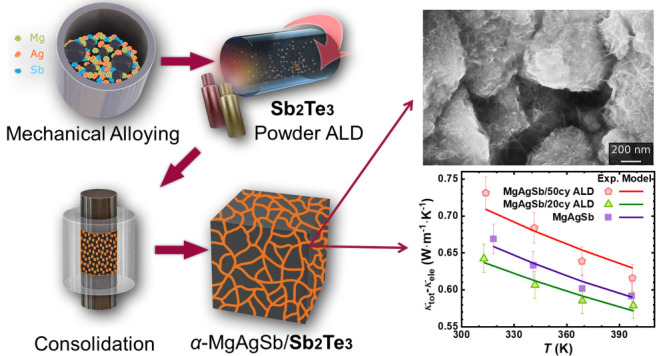

## 1 Introduction

Thermoelectric (TE) materials convert heat directly into electricity, offering a promising route for waste heat recovery with advantages such as scalability, mechanical simplicity, and long-term reliability.^1,2^ Their efficiency is determined by the dimensionless figure of merit: *zT* = *S* ^2^ *σT*/*κ*_tot_, where *S* is the Seebeck coefficient, *σ* is the electrical conductivity, *T* is the absolute temperature, and *κ*_tot_ is the total thermal conductivity.^3^ *κ*_tot_ consists of lattice (*κ*_lat_) and electronic (*κ*_ele_) contributions. Achieving a high *zT* therefore requires simultaneously maximizing the power factor (PF = *S* ^2^ *σ*) while minimizing *κ*_tot_. However, due to the intercorrelation of the transport parameters, optimizing the TE performance is relatively complex. Because the electrical properties involving *S*, *σ* and *κ*_ele_ are strongly coupled, improving one of these three parameters will result in compensation for other parameters that impede progress toward improving TE performance. Minimizing the total thermal conductivity, particularly for the only independent TE parameter of *κ*_lat_, was deemed to be a promising strategy by seeking intrinsically low thermal conductivity materials and lattice anharmonicity.^4^

Among low *κ*_lat_ materials, α-MgAgSb has emerged as a leading candidate for near room temperature TE applications. It combines intrinsically suppressed lattice thermal conductivity with earth-abundant, nontoxic elements and a competitive *zT* > 1.0 below 550 K.^5^ These advantages make α-MgAgSb a strong alternative to Bi_2_Te_3_, though historically dominant in commercial TE applications, relying on scarce and toxic tellurium resources.^6,7^ Recent studies have explored various strategies to enhance the α-MgAgSb performance, focusing on doping and enhancing phase purity.^5,8–13^ However, a fundamental challenge of α-MgAgSb on how to simultaneously increase *S* while further decreasing the thermal conductivity to achieve higher *zT* has remained unexplored. One promising and widely investigated approach corresponds to the interface modification of TE materials with the goal of decreasing thermal conductivity and increasing *S*, resulting in great success in enhancing *zT* values.^14^ Introducing a secondary phase either as dispersed particles or as a conformal coating can create dense interfaces that effectively scatter midwavelength phonons, thereby lowering *κ*_lat_.^15^ Moreover, at the electronic level, such interfaces can act as energy filters, preferentially scattering low-energy carriers and thus enhancing *S* without severely degrading *σ*. Compared to nanoprecipitation strategies employed in traditional systems (e.g., PbTe,^16^ AgSbTe_2_ ^17^), controlled interface modification via a continuous second phase coating allows for better uniformity in phase distribution and interface characteristics, leading to more reliable improvements in TE performance. The main challenge, however, lies in achieving uniform and precisely controlled coatings on TE powders. Atomic layer deposition (ALD) offers a unique solution, as it enables conformal, layer-by-layer growth of ultrathin films with angstrom-level thickness control, even on powders with complex morphologies.^18,19^ In powder ALD, this precision translates to core–shell particles, in which the coating remains at grain boundaries after densification, yielding well-defined interfaces in the bulk material. Powder ALD has already demonstrated significant enhancements in Bi_2_Te_3_,^20^ CoSb_3_,^21^ and ZrNiSn^22^ systems by suppressing thermal conductivity and tuning carrier transport.

In this work, we extend powder ALD to α-MgAgSb using Sb_2_Te_3_ as a secondary phase coating. Oxide coatings, common in prior powder ALD studies, are unsuitable for α-MgAgSb due to its complex phase equilibrium and the high mobility of Mg and Ag atoms, which promote parasitic oxide formation and degrade electrical conductivity.^23^ In contrast, Sb_2_Te_3_ is an excellent candidate: it exhibits high thermoelectric power factor,^24^ has a compatible thermal expansion coefficient with α-MgAgSb,^25,26^ and nucleates readily on Sb-containing surfaces, ensuring uniform film growth during ALD.^27–30^ Here, we demonstrate the feasibility of nonoxide powder ALD coatings on α-MgAgSb. To minimize the possible oxidation of the powder, the synthesis, as shown in Figure 1, is duplicated inside an inert atmosphere. Our results show that conformal Sb_2_Te_3_ coatings can reduce lattice thermal conductivity and highlight a pathway for interface engineering in α-MgAgSb beyond conventional oxide-based powder ALD. This work establishes a foundation for broader application of semiconductor and metallic ALD coatings in TE materials, opening opportunities for further optimization of interfacial transport.

**Figure 1:**
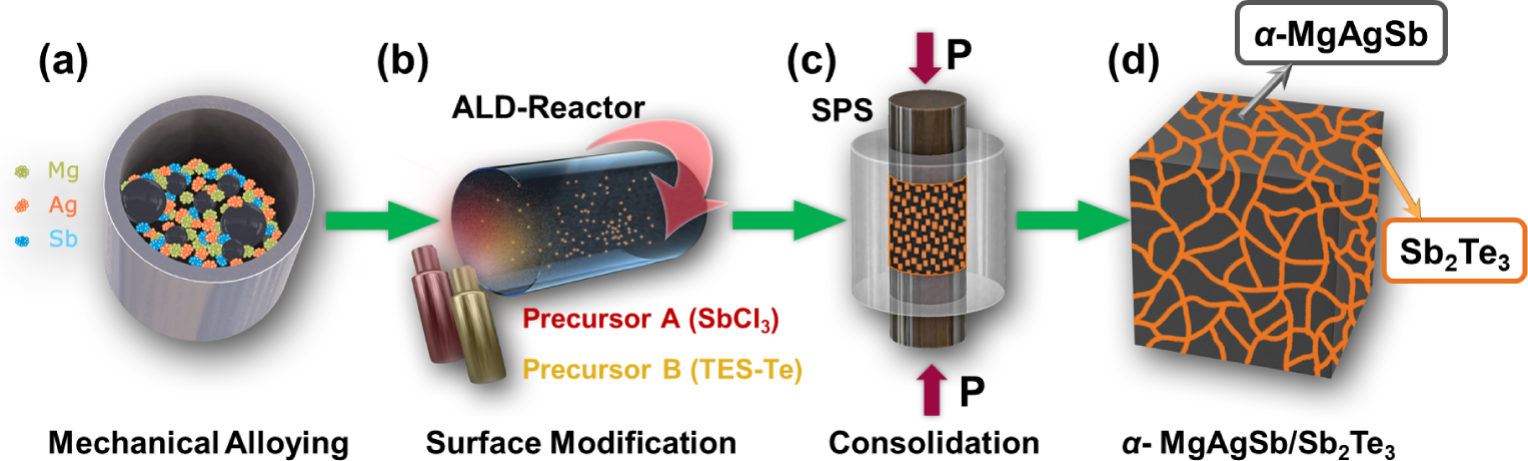
Schematic of the fabrication route. (a) Mechanical alloying of the Mg, Ag, and Sb powders, (b) Sb_2_Te_3_ ALD coating of the matrix powders, (c) consolidation via SPS and (d) final resulting α-MgAgSb bulk with Sb_2_Te_3_ strategically located at the former particle interfaces.

## 2 Results and Discussion

To properly assess the effect of the ALD coating layers on the α-MgAgSb powders, the film’s composition and growth profile were first characterized. For this investigation, Quartz Crystal Microbalance (QCM) data was recorded during the ALD process and is shown in Figures 2a and S1. It showcases a mass gain of Sb_2_Te_3_ of 1.21 ng/cm^–2^ per cycle or 0.18 Å per cycle (density of the Sb_2_Te_3_ films 6.50 g/cm^–3^ )^31^ at 358 K, which is comparable to the 0.17 Å per cycle reported at 363 K by Pore et al.^27^ The initial rapid mass increase in Figure S1 shows the saturation of the whole QCM monitoring surface, followed by a consistent and linear monolayer formation. The observed homogeneous mass increase proportional to cycle numbers indicates a complete self-limiting ALD profile without parasitic Chemical Vapor Deposition (CVD),^32^ evidencing an excellent thickness controllability at the subnanoscale. The X-ray diffraction (XRD) data in Figure 2b show that the deposited film can be indexed by the structure model of rhombohedral Sb_2_Te_3_ with the space group *R*-3*m* ^33^. Besides some small reflections of the silicon substrate, no other signals that correspond to reaction precursors or byproducts left on the substrate were detected. The deviation of the relative XRD intensities observed from the standard reference is a consequence of texture and measurement geometry. The ALD-grown Sb_2_Te_3_ exhibits a pronounced preferred orientation compared to the reference powder pattern, which is also consistent with the characteristic plate-like morphology^34^ observed in the Scanning Electron Microscope (SEM) images in Figure 2c,d.

**Figure 2:**
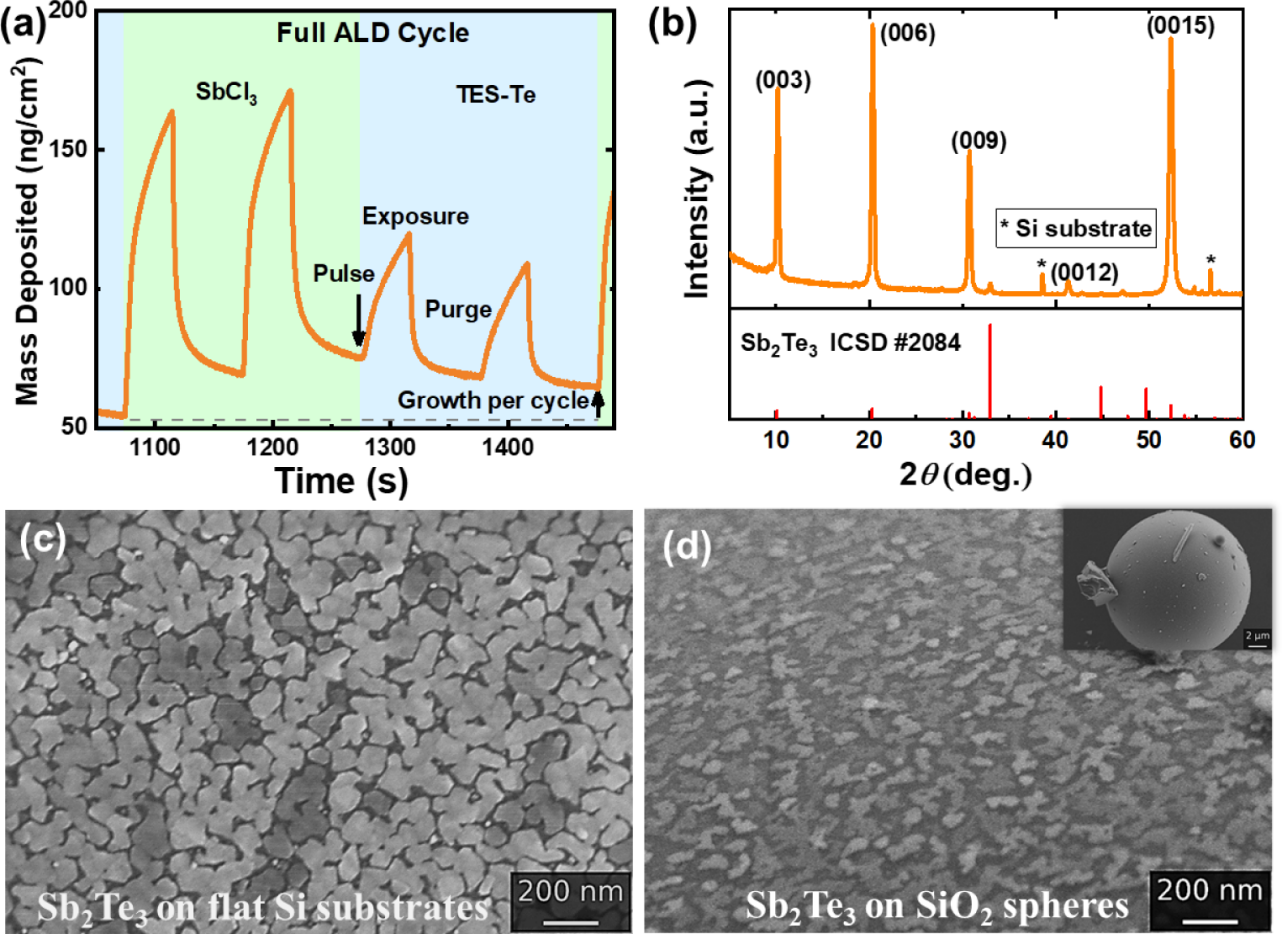
(a) QCM data showcasing the linear growth rate of the Sb_2_Te_3_ layer; (b) XRD data of the crystalline Sb_2_Te_3_ deposition,^33^ (c) SEM image of the Sb_2_Te_3_ deposition on flat Si substrates, (d) SEM image of the Sb_2_Te_3_ deposition on SiO_2_ spheres (inset: full SiO_2_ sphere for reference).

The growth of a crystalline Sb_2_Te_3_ film indicates no secondary phases, eliminating the need for any postdeposition annealing step. After the crystallinity of the ALD film was confirmed, α-MgAgSb powders were coated for 10, 20, 30, and 50 cycles of Sb_2_Te_3_. While we can characterize the growth per cycle on Si substrates by the QCM, the different surface chemistries of α-MgAgSb powders could lead to a modification of the layer formation mechanism. Therefore, we address the coated powders by their number of coating cycles, and not by the equivalent thickness obtained by the same number of cycles on a Si surface.

When the *α-*MgAgSb powders are coated and consolidated into a pellet, we observe the formation of plate-like crystalline growths at their fracture surface, which increase according to the cycle number (Figures 3a,b and S2). These crystallites closely resemble the growth profile of a delaminating ALD film, a type of thin film growth behavior present when there’s poor adhesion of the layer to the substrate, which has already been reported for Sb_2_Te_3_ ALD.^34^ The observed difference in crystalline growth morphologies between the model substrates and the MgAgSb powder reflects the differences in the surface chemistry. During sintering, thermal and mechanical stresses at particle contacts promote partial delamination and reorientation of the Sb_2_Te_3_ layer, leading to vertically protruding nanosheets as seen on the boundaries in the MgAgSb-50 cycles sample. To reassure that the coating does not affect the α-MgAgSb matrix, XRD measurements were performed and the resulting diffraction patterns are displayed in Figure 3c. The purity of α-MgAgSb remains chemically and thermally unchanged during the process. Images from Scanning Electron Microscopy utilizing a Backscatter electron detector (SEM-BSD) (Figure S3) reveal a predominantly uniform contrast for samples across increasing coating cycles, with only sparse bright or dark regions that can be attributed to minor impurity phases or local compositional fluctuations already present in the pristine material. Within the spatial resolution of SEM-BSD, these inhomogeneities show no systematic change with increasing Sb_2_Te_3_ coating cycles, indicating that the ALD process does not introduce additional impurity segregation. Since the presence of Sb_2_Te_3_ is not conclusive by XRD, X-ray Photoelectron Spectroscopy (XPS) was used to evaluate the presence and binding state of tellurium on the sample with the most promising decrease in thermal conductivity (MgAgSb-20 cycles of Sb_2_Te_3_). The core-level spectra of Mg 1s, Ag 3d, Sb 3d, Te 3d, O 1s, and C 1s are depicted in the survey spectra in Figure 3d. The presence of the O 1s and C 1s peaks can be due to atmospheric contamination during transfer to the XPS device. On the high-resolution XPS spectra of the coated sample in the Te region shown in the inset figure of Figure 3d, the peaks exhibited at binding energies of ∼573.5 eV and ∼586.5 eV were identified as those of Te 3d5/2 and Te 3d3/2, respectively, and confirm the presence of Te after consolidation.^35,36^ The high-resolution XPS spectra corresponding to Mg 1s, Ag 3d, and Sb 3d for the pristine and coated samples are displayed in Figure S4. Based on the XRD and XPS analyses, there is no evidence of Sb_2_Te_3_ coatings diffusing into the MgAgSb matrix and forming secondary phases. Hence, the coating was deposited on the powder surfaces and did not migrate or react during SPS consolidation.

**Figure 3:**
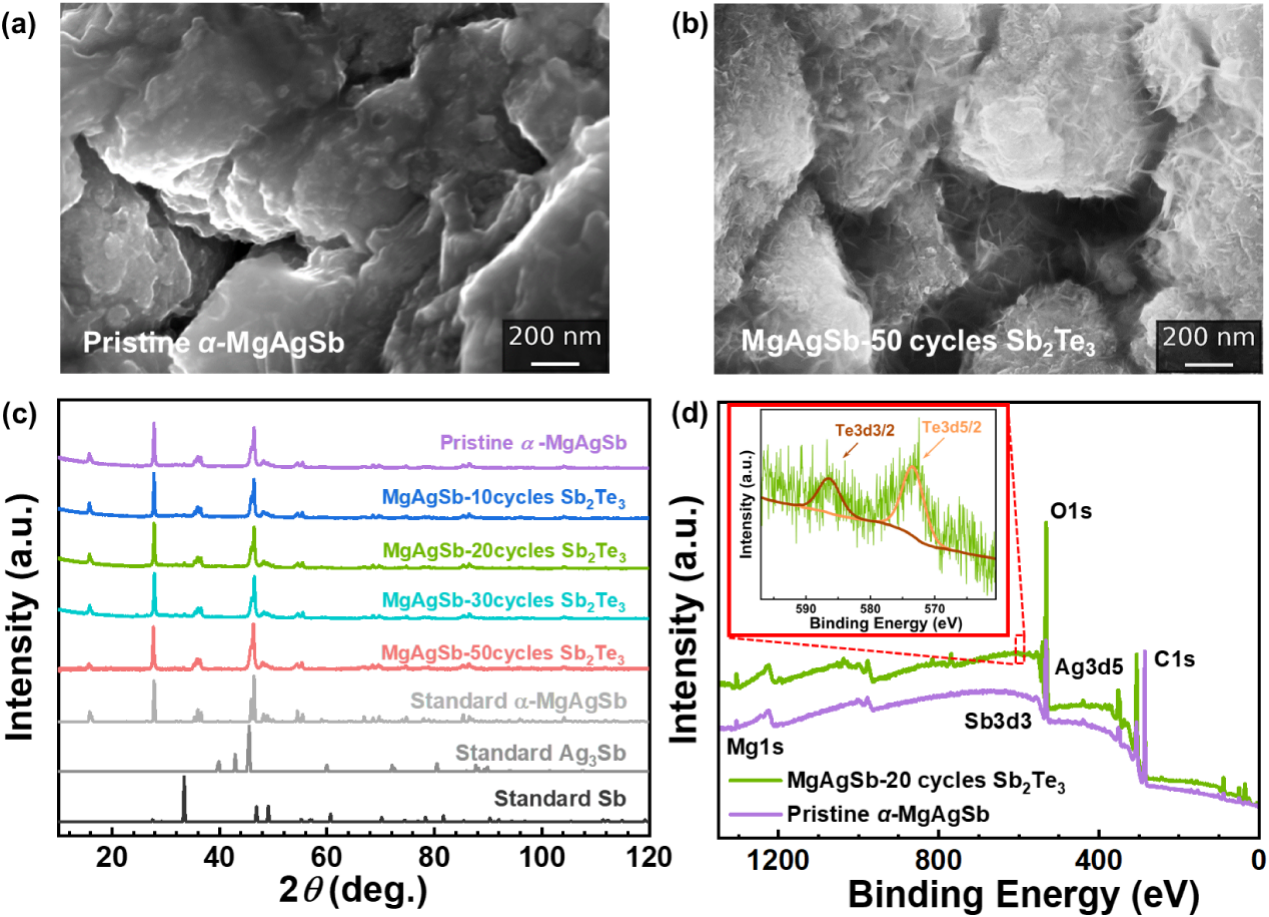
SEM fractograms of (a) pristine α-MgAgSb, (b) MgAgSb-50 cycles Sb_2_Te_3_, (c) XRD of the pristine and coated α-MgAgSb compared to reference ICSD data,^37^ and (d) XPS survey of the pristine α-MgAgSb and MgAgSb-20 cycles Sb_2_Te_3_, insert figure with high resolution XPS of MgAgSb-20 cycles Sb_2_Te_3_ on the region corresponding to the Te 3d3/2 and Te 3d5/2 peaks.

Building on the structural findings, we subsequently evaluated the influence of the coating on the thermoelectric transport properties. The thermoelectric properties of α-MgAgSb are found to be highly sensitive to surface conditions, particularly to the presence and integrity of the applied coating. Exposure to oxygen during sample transfer to the ALD reactor under noninert conditions was identified as a primary source of degradation in thermoelectric performance (Figure S5). This oxidation leads to a marked reduction in the electrical transport properties and a concomitant decrease in the overall *zT*. Therefore, only samples processed entirely within the ALD reactor under an inert atmosphere are considered for further analysis.

For the coated α-MgAgSb samples, the electrical conductivity decreases nonmonotonically with increasing coating thickness (Figure 4a). This trend can be explained by the nature of the ALD growth profile. At low coating cycles (pristine to 10 cycles), the electrical conductivity remains in a similar range as isolated Sb_2_Te_3_ islands start to form on the powder surfaces. As the coating increases to 20 cycles, a marked decrease is noted due to the formation of extended coatings. These heterointerfaces act as scattering centers for charge carriers, causing the observed decrease in conductivity. This is consistent with the carrier mobility data in Table S1 and Figure 4d, which show a progressive reduction with coating cycling. When more coating cycles (30 to 50) are deposited, cracks form in the coated areas, leading to less efficient coverage and a lower heterointerface density, thereby reducing their detrimental effect on electrical conductivity. This behavior is consistent with established mechanisms observed in other interface-engineered thermoelectric materials and demonstrates the balance between reduced phonon scattering and increased charge carrier scattering.^38^ The Seebeck coefficient remains in a similar range across all samples (Figure 4b), suggesting minimal changes in carrier concentration, consistent with the Hall measurements results (Table S1). The slight rise in the Seebeck coefficient for the sample coated with 10 cycles at near room temperature is within the uncertainty on the measuring system (7%). Consequently, these changes in the charge transport properties result in a PF that initially increases at 10 cycles of Sb_2_Te_3_ coating due to the slight increase in the Seebeck coefficient, but decreases for higher coating thicknesses following the electrical conductivity trend (Figure 4c). The Hall carrier measurement further confirms the mobility decrease with coating cycles (Figure 4d).

**Figure 4:**
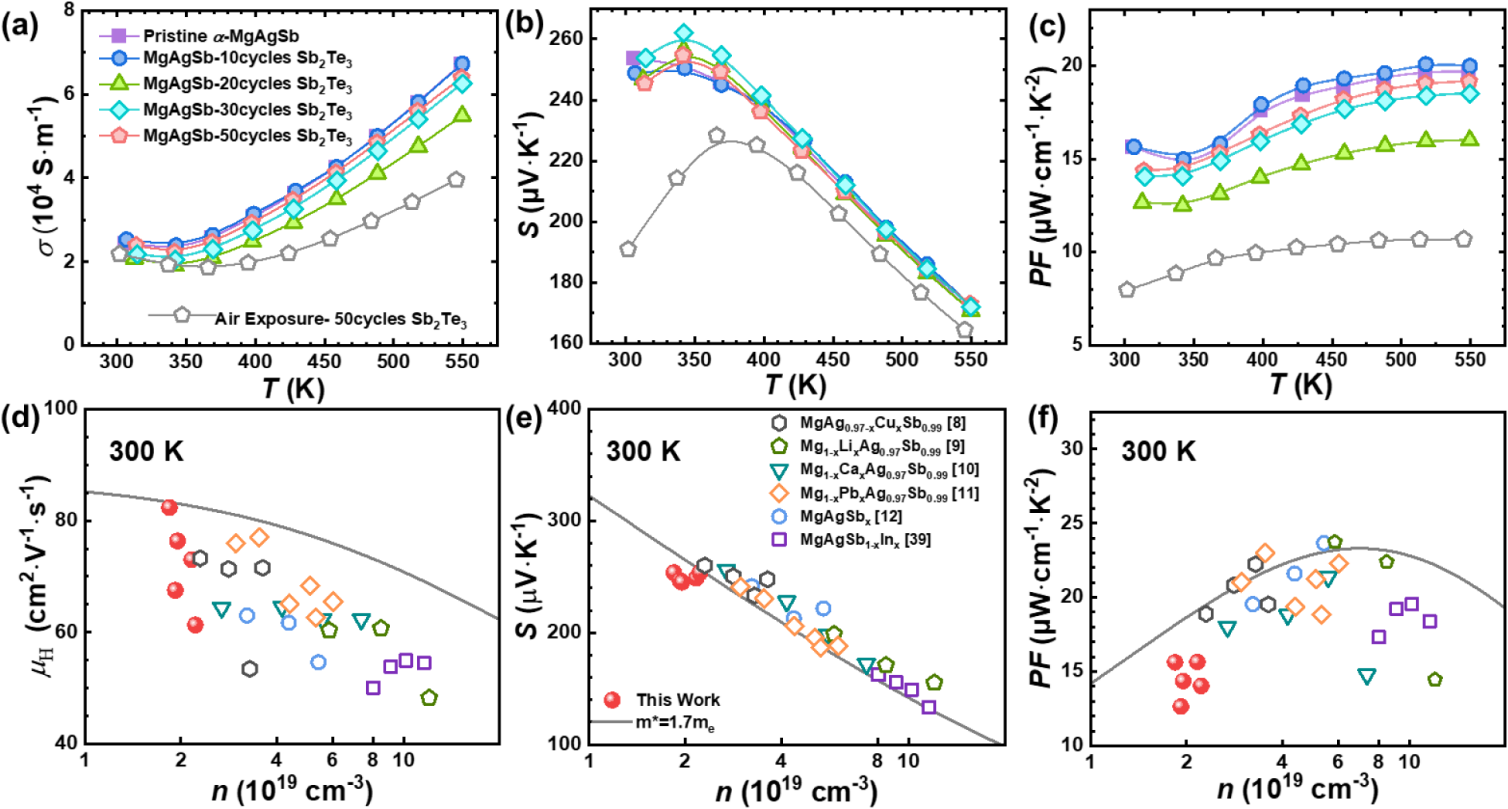
Temperature dependence of the (a) electrical conductivity, (b) Seebeck coefficient, (c) power factor, (d) relation of the carrier mobility with the Hall carrier concentration, (e) Pisarenko plot, (f) relation of the power factor with the carrier concentration calculated using the SPB model (gray line, m* = 1.7 *m_e_*) and experimental data in this work, compared with reported data at room temperature.^8–12,39^

To gain deeper insight into the charge transport behavior, the Single Parabolic Band (SPB) model was applied to assess the coated samples’ optimal carrier concentration and Seebeck coefficient.^40,41^ Theoretically, within the Single Parabolic Band (SPB) model, increasing the Hall carrier concentration shifts the Fermi level deeper into the band and thus reduces the Seebeck coefficient, giving rise to the Pisarenko relation.^40^ In Figure 4e, the experimental Seebeck coefficients of all samples fall onto a single Pisarenko curve, indicating that they retain similar Seebeck coefficients and effective masses. Minor variations from the SPB model are due to experimental uncertainties and measurement errors, however, the obtained values align with values given by literature.^8–12,39^ This demonstrates that the Sb_2_Te_3_ coating does not appreciably modify the Hall carrier concentration of the α-MgAgSb matrix, as detailed in Table S1. Instead, the main effect of the coating is to introduce additional grain-boundary and interfacial scattering, which reduces carrier mobility and electrical conductivity (Figure 4a,d) without altering the fundamental relationship between the Seebeck coefficient and the Hall carrier concentration.

However, the carrier mobility decreases more sharply with carrier concentration than predicted by the SPB model (Figure 4d). This decline is due to additional scattering mechanisms not assessed adequately by the SPB, as it assumes a single main scattering mechanism.^42^ The reduced mobility compared to the pristine sample is interpreted as the direct consequence of extra grain boundary scattering or interfacial defects introduced by the Sb_2_Te_3_ coating layers. The reduction in PF of the samples with more than 10 cycles is attributed to the lowered electrical conductivity (due to decreased mobility), even though the Seebeck coefficient remains almost unchanged (Figure 4c,f).

Notably, the thermal conductivity decreases with increasing Sb_2_Te_3_ coating thickness, consistent with enhanced phonon scattering introduced by the coating layer. As shown in Figure 5a, the total thermal conductivity of the materials decreases near room temperature from ∼0.8W/mK for the pristine material to ∼0.7W/mK at 300 K for MgAgSb with 20 cycles of Sb_2_Te_3_ coating. However, beyond 20 cycles, there is a slight rebound in thermal conductivity. This rise can be due to the coating becoming too thick, cracking and exposing the neighboring grains during sintering, hindering the coating effect. Previous studies in the literature have reported a similar phenomenon by which the growth of a coating layer at the particle surface beyond a specific thickness becomes detrimental, due to the change in the film morphology.^14,20^ When the electronic contribution is subtracted from the total thermal conductivity, it is observed that the lattice thermal conductivity keeps the same trend with cycling as the total thermal conductivity, confirming that the reduction in *κ*_tot_ is primarily due to increased phonon scattering rather than changes in electronic thermal conductivity (Figure S6).

**Figure 5:**
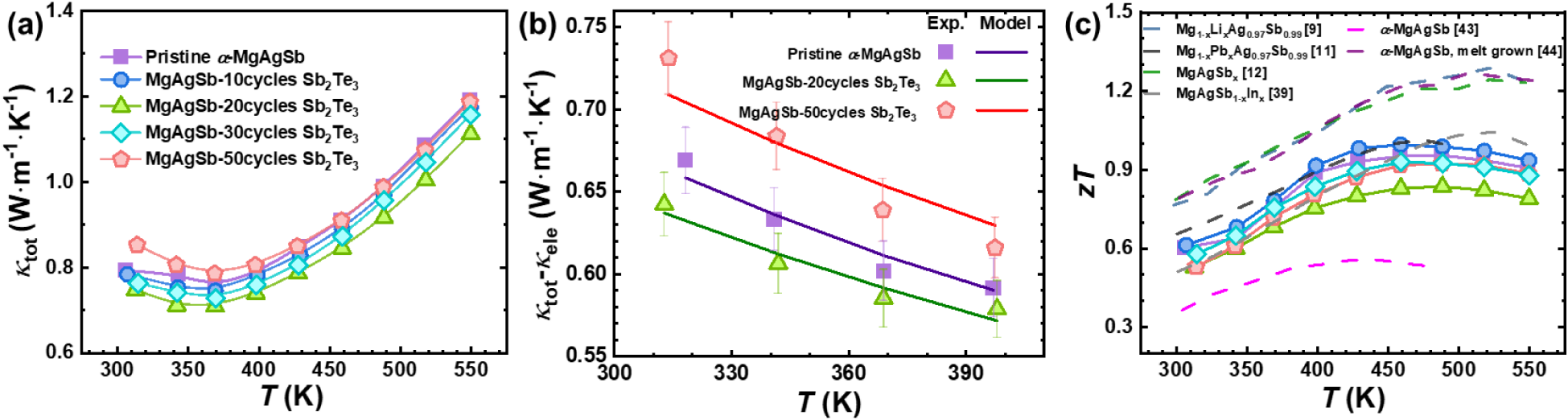
(a) Temperature dependence of total thermal conductivity, (b) Debye Callaway model of the lattice thermal conductivity, and (c) temperature dependence of the *zT* compared to literature values.^9,11,12,39,43,44^

To elucidate the phonon scattering mechanisms responsible for this behavior, the Debye-Callaway model was used to fit the lattice thermal conductivity data (Figure 5b). Four fitting parameters were used to assess the four dominant scattering modes (point defects, nanoparticles and inclusions, grain boundaries, and Umklapp scattering).^45,46^ Details of the model are provided in the Supporting Information. The evolution of the fitting parameters reveals the dominant phonon scattering processes is influenced by the coating thickness. Among all samples, the one with 20 cycles of Sb_2_Te_3_ coating exhibits the highest point defect scattering fitting coefficient (A = 6.4·10^–40^ s^3^), nanoparticle scattering coefficient (C = 5.0·10^–30^ m^3^s^–3^), and a grain boundary scattering coefficient 1 order of magnitude higher when compared to the pristine sample (D = 64.5·10^–6^), while simultaneously showing the lowest Umklapp scattering coefficient (B = 1.0·10^–8^ m^3^K^–1^s^–2^). This fitting explains the lower lattice thermal conductivity in MgAgSb-20 cycles Sb_2_Te_3_, as the scattering is more dependent on grain boundary effects. The increased point defect scattering and nanoparticle scattering coefficients suggest enhanced mass fluctuation and interfacial mismatch with the coating layer, possibly due to a higher density of interfaces or inclusions introduced at 20 ALD cycles. This finding is further supported by the significantly elevated grain boundary scattering parameter, which reflects a substantial reduction in grain size compared with the pristine sample. Compared to the other samples, MgAgSb-20 cycles Sb_2_Te_3_ shows the most pronounced deviation in scattering behavior, indicating an optimal coating thickness that strongly impacts phonon transport. This reduction of the lattice thermal conductivity with the coating thickness supports the presence of enhanced phonon scattering due to heterointerfaces at the particle boundary and validates the effectiveness of the coating. The adjusted fitting values obtained for all samples are summarized in Table S2. While the decrease in lattice thermal conductivity is not dramatic, it should be interpreted in the context of α-MgAgSb, which already exhibits intrinsically very low *κ*_lat_. In this ultralow *κ*_lat_ regime, additional interface scattering can only generate limited relative reductions before electrical transport is strongly compromised. The decrease observed for MgAgSb with 20 ALD cycles of Sb_2_Te_3_, therefore, represents a nontrivial change as well as a shift in the predominant scattering mechanism. This is confirmed by the pronounced increase in the grain boundary scattering coefficient in the Debye Callaway fitting parameter. The present coating thicknesses reflect a deliberate compromise between thermal and electrical transport. The interplay between these electrical and thermal effects collectively determines the overall thermoelectric performance. As shown in Figure 5c, the *zT* of MgAgSb-10 cycles Sb_2_Te_3_ increases over the entire temperature range, driven by the reduction in lattice thermal conductivity and slight Seebeck coefficient increase despite the modest reduction in electrical conductivity. For deposition cycling over 20 cycles of Sb_2_Te_3_, the detrimental effects of the coating on the electrical properties hinder the material’s performance. Furthermore, the effective mass and carrier concentration remain largely unchanged as shown by the Pisarenko plot in Figure 4e, suggesting that improvements in *zT* must arise from modifications in the thermal transport properties, and validating the role of the ALD process in enhancing the phonon scattering. Notably, we have been able to obtain *zT* values comparable to those of doped α-MgAgSb, without actually introducing dopants into the matrix. These findings demonstrate that interface engineering via powder ALD can serve as an effective alternative to doping for enhancing thermoelectric performance. Further optimization of the coating thickness and carrier concentration could yield even higher *zT* values, offering a promising route toward high-efficiency, compositionally stable α-MgAgSb-based thermoelectric materials.

## 3 Conclusion

In this study, we demonstrated the use of powder ALD to deposit Sb_2_Te_3_ coatings on the surface of α-MgAgSb powders as a way to reduce the lattice thermal conductivity of the subsequently sintered material. By optimizing the ALD process in an inert atmosphere, we successfully deposited uniform, crystalline Sb_2_Te_3_ layers without compromising the phase purity of the α-MgAgSb matrix. Our results show that the coating effectively alters phonon scattering mechanisms, particularly at the grain boundaries, leading to a noticeable decrease in the lattice thermal conductivity. A minimum lattice thermal conductivity is reached at 20 cycles of Sb_2_Te_3_ ALD coating, achieving a 10% reduction when compared to the pristine material. Although the electrical conductivity and carrier mobility declined slightly due to increased carrier scattering, the carrier concentration remained stable, suggesting that the electronic band structure of α-MgAgSb was largely unaffected. The Debye-Callaway model supported these findings, indicating that enhanced grain boundary scattering was primarily responsible for the reduction in thermal conductivity. While the minimum lattice thermal conductivity is achieved after 20 cycles of Sb_2_Te_3_ coating, the best thermoelectric performance is reached at MgAgSb-10 cycles Sb_2_Te_3_, where the electrical transport is less affected and the *zT* is enhanced over the entire temperature range. This work highlights the potential of oxygen-free ALD coatings for air-sensitive thermoelectric materials, offering a promising alternative to the previous oxide-based approaches. It also demonstrates the tunability of the thermoelectric properties via the modification of the coating thickness. Overall, our findings provide a new avenue for interface engineering in complex thermoelectric materials as a complementary strategy to traditional doping.

## 4 Methods

### 4.1 Materials Synthesis

High-purity Mg (shards, 99.8%, AlfaAesar), Ag (powder, 99.9%, HMW Hauner GmbH), and Sb (shards, 99.999%, MaTeck) were weighed out in the atomic ratios of MgAg_0.97_Sb_0.99_ (denoted as α-MgAgSb in the text). 8 g total of weighed elements were loaded together into a hardened steel ball-milling jar inside a glovebox under an argon atmosphere with oxygen and water levels below 1.0 ppm and then ball-milled for 20 h using High Energy Ball Milling (SPEX 8000D High Energy Ball Mill), on intervals of 5, 10, and 5 h, manually loosening the powders from the ball-milling jar walls in between.

The Sb_2_Te_3_ ALD thin films were deposited using an ALD hot wall steady flow reactor (Arradiance GEMStar-XT) installed inside a glovebox under an argon atmosphere with an oxygen and water level below 1.0 ppm. In order to evaluate the effect of air exposure on the powders, a duplicate of all samples was also prepared in a Veeco Savannah S200 mounting a dome lid with a Particle Coating adapter tube outside of the inert atmosphere. Bis­(triethylsilyl) tellurium ((Te­(SiEt_3_)_2_), synthesized according to a method previously described in literature,^47^ 99.8%) and antimony trichloride (SbCl_3_, Merck, ACS reagent, ≥99.0%) were used as ALD precursors and heated to 350 and 333 K, respectively. The ALD deposition took place at 358 K. High purity Argon was used as a carrier gas, maintaining a steady flow of 80 sccm in the case of the Arradiance GEMStar-XT reactor and 40 sccm for the Veeco Savannah S200 in order to accommodate the different reactor volumes. The optimized pulse, exposure, and purge times for one ALD cycle were 5/35/60×2//5/35/60×2s (((Te­(SiEt_3_)_2_ pulse/exposure/purge ×2//SbCl_3_ pulse/exposure/purge ×2). A scheme detailing the sections of an ALD cycle can be seen in Figure 2a. A double precursor pulsing was used to ensure appropriate vapor pressure and even coating throughout the entire powder surface.

Si substrates with a 100 nm thermal SiO_2_ layer were cleaned with acetone and isopropanol in an ultrasonic bath for 15 min, respectively, dried with a N_2_ gun, introduced in an ozone cleaner for 5 min, and used to monitor the ALD layer crystallinity, morphology, and growth rate. After assessing the quality of the deposition on flat substrates, SiO_2_ spheres (10 μm radius) were coated using a custom-made particle coating cylinder rotating at 15 rpm to observe the deposition characteristics on high surface area substrates. Si and SiO_2_ substrates were coated with up to 400 cycles of Sb_2_Te_3_ in order to obtain a coating that was thick enough for characterization. Finally, the α-MgAgSb powder after ball milling was coated in 1.2 g batches with 10, 20, 30, and 50 cycles of Sb_2_Te_3_. The coated α-MgAgSb powders were subjected to Spark Plasma Sintering (SPS, AGUS-PECS SPS-210Sx, SUGA Co.) inside a glovebox under vacuum conditions, sintered in a graphite 10 mm wide die under a pressure of 45 MPa at 553 K for 5 min, and then treated at 523 K for 20 min.

### 4.2 Characterization and Measurements

The ALD deposited Sb_2_Te_3_ thin films on Si substrates were analyzed for phase purity and crystal structure by X-ray diffraction (XRD, Bruker D8 Advance, Co radiation), and their morphology was analyzed by Scanning Electron Microscopy (SEM Sigma 300, Zeiss). The ALD growth rate was monitored using an Inficon Front Load Single Sensor connected to an Inficon STM-2 Deposition Monitor, with 6 MHz Gold Coated Quartz Monitor Crystals. The phase purity and crystal structure of the sintered samples were examined by X-ray diffraction (XRD, Bruker D8 Advance, Co radiation), and their microstructures were analyzed by Scanning Electron Microscopy (SEM Sigma 300, Zeiss). The lattice parameters and crystallite size of the pristine sample were obtained by a Rietveld analysis with the FullProf program of XRD data obtained on a Stoe Stadi P (equipped with a curved Ge (111) primary beam monochromator, a Mythen 1 K detector (Dectris) on a flat sample in transmission geometry with Cu K_α1_ radiation (λ = 1.5406 Å). Before the measurement, a piece of pellet was powdered in an agate mortar to overcome the strong preferred orientations of the pellet. The tellurium content and oxidation state of the coated powders were analyzed via X-ray photoelectron spectroscopy (XPS, ESCALAB 250Xi, Thermo Scientific).

The temperature-dependent Seebeck coefficient (*S*) and electrical resistivity (ρ) were measured by the standard four-probe method (LSR-3, Linseis). The temperature-dependent thermal diffusivity (α) was measured by a laser flash method under a helium atmosphere (LFA 1000, Linseis). The density (*D*) of the samples was measured by the Archimedes method, and the heat capacity (*C*_p_) was obtained from previous reports.^39^ The *κ*_tot_ was calculated according to the relation *κ*_tot_ = α*DC*_p_. The electrical contribution to the thermal conductivity was obtained by the Wiedemann–Franz law, where the Lorentz number was approximated by $L=1.5+e^{[\frac{-\left|S\right|}{116}]}$ .^48^ The longitudinal and shear sound velocities were measured using a UT340 Pulser-Receiver with 20 MHz X-ray Olympus transducers at room temperature. The Hall concentration (*n*_*H*_) was measured using the Hall-bar method (PPMS, Quantum Design) under a ± 9 T magnetic induction at room temperature. The Hall mobility (μ_*H*_) was calculated as *μ*_*H*_ = σne, where *e* is the elementary charge. The relation of the electric properties on the carrier concentration was calculated utilizing the Single Parabolic Model (SPB).^40,41^ The Debye-Callaway model was used to further understand the underlying phonon scattering mechanisms that govern the lattice thermal conductivity.^46^ The equations used for the SPB and the Debye-Callaway Model are given in the Supporting Information (eqs S1–S13).

## Supplementary Material



## Notes

Additional experimental details, materials, and methods; QCM data, SEM images, XPS spectra, XRD refinement, thermoelectric characterization, and details on the SPB and the Debye-Callaway Model used (PDF)
