# Supplementary material for: Reduced Lattice Thermal Conductivity in Thermoelectric α‑MgAgSb via Sb2Te3 Powder Atomic Layer Deposition
Source: ACS Appl Mater Interfaces. 2026 Feb 6;18(6):10611–9. doi: 10.1021/acsami.5c23388 (PMC12926952; doi:10.1021/acsami.5c23388)
Supplement: Supplementary file 1 [file am5c23388_si_001.pdf]

Supporting information

# Reduced lattice thermal conductivity in thermoelectric $\alpha$-MgAgSb via $Sb_2Te_3$ powder atomic layer deposition

Irene Garcia Santamaria [a,b], Amin Bahrami [a], Angelika Wrzesinska-Lashkova [a,c], Jaroslav Charvot [d], Andrei Sotnikov [a], Lars Giebeler [a], Yana Vaynzof [a,c], Filip Bures [d], Pingjun Ying [a*], Kornelius Nielsch [a,b,e*]

[a] *Leibniz Institute for Solid State and Materials Research Dresden, 01069 Dresden, Germany*

[b] *Institute of Materials Science, Technische Universität Dresden, 01062 Dresden, Germany*

[c] *Chair for Emerging Electronic Technologies, Technische Universität Dresden, 01187 Dresden, Germany*

[d] *Institute of Organic Chemistry and Technology, Faculty of Chemical Technology, University of Pardubice, 53210 Pardubice, Czech Republic*

[e] *Institute of Applied Physics, Technische Universität Dresden, 01062 Dresden, Germany*

* Correspondence to: e-mail: p.ying@ifw-dresden.de (Pingjun Ying), k.nielsch@ifw-dresden.de (Kornelius Nielsch)

Keywords: Thermoelectric, energy harvesting, $\alpha$-MgAgSb, Powder Atomic Layer Deposition, $S_2Te_3$

**Restructured Single Parabolic Model**

A restructured Single Parabolic Band Model (SPB) proposed by J. Zhu et al.[1] was used to obtain the Pisarenko plot and the carrier mobility dependence with carrier concentration, by which the effective mass $m*$, intrinsic mobility $\mu_0$, weighted mobility $\mu_{wt,}$ and maximum PF can be calculated as:

$$\left(\frac{m^*}{m_e}\frac{T/K}{300}\right)^{3/2} = \frac{n}{n_{m,0}}\left[exp\left(\frac{S}{S_0}-2\right)-0.1455\right](S1)$$

$$\mu_0 = \mu\left[1+\left(\frac{m^*}{m_e}\frac{T/K}{300}\right)^{-3/2}\frac{n}{2n_{m,0}}\right]^{1/3} \quad (S2)$$

$$\mu_{WT} = \mu_0\left(\frac{m^*}{m_e}\frac{T/K}{300}\right)^{3/2}, \mu_W = \mu_0\left(\frac{m^*}{m_e}\right)^{3/2} \quad (S3)$$

$$PF_{max}/\mu W cm^{-1}\,K^{-2} = 0.1212\,\mu_{WT}/cm^{-2}\,V^{-1}s^{-1}(S4)$$

And therefore, the SPB can be restructured and numerically solved as:

$$n = n_{m,0}\left(\frac{m^*}{m_e}\frac{T/K}{300}\right)^{3/2}\cdot n_r(S5)$$

$$\sigma = n_{m,0}q\mu_{WT}\cdot\left(\frac{m^*}{m_e}\frac{T/K}{300}\right)^{3/2} n_r\left(1+\frac{n_r}{2}\right)^{-1/3} \quad (S6)$$

$$S = S_0\cdot ln\left(1.075+\frac{e^2}{n_r}\right)(S7)$$

$$L = L_0\cdot\left\{2+\frac{\pi^2/3-2}{[1+(2\pi/n_r)^{3/2}]^{2/3}}\right\}(S8)$$

$$PF = {S_0}^2 n_{m,0}q\mu_{WT}\cdot n_r\left(1+\frac{n_r}{2}\right)^{-1/3} ln^2\left(1.075+\frac{e^2}{n_r}\right)(S9)$$

Were $n_{m,0} = 2.5094\cdot 10^{19}cm^{-3}$, $S_0 = 86.1733\,\mu VK^{-1}$, $L_0 = 0.7426\cdot 10^{-8}W\Omega K^{-2}$ , and $q = 1.6021766341\cdot 10^{-19}C$.

**Debye-Callaway model**

The Debye-Callaway model was used to better understand the contributions to the lattice thermal conductivity and the scattering mechanisms. The lattice thermal conductivity can be expressed as:

$$\kappa_L = \frac{k_B}{2\pi^2 \nu}\left(\frac{k_B T}{\hbar}\right)^3 \int_0^{\theta_D/T} \frac{x^4 e^x}{\tau^{-1}(e^x - 1)^2} dx \#(S10)$$

Where $x = \hbar\omega/k_B T$ is the reduced phonon frequency, $\omega$ is the phonon frequency, $\nu$ is the average phonon group velocity calculated from the transverse and shear modes as[2,3]:

$$\nu = \left(\frac{1}{3}\left[\frac{1}{\nu_s^3} + \frac{2}{\nu_t^3}\right]\right)^{-1/3} \quad (S11)$$

$\theta_D$ is the Debye temperature obtained from[2]:

$$\theta_D = \frac{h}{k_B}\left[\frac{3N}{4\pi V}\right]^{1/3} \quad (S12)$$

, and $\tau^{-1}$ is the total phonon scattering relaxation time. The phonon scattering relaxation time can be further expressed as the combination of the different scattering mechanisms through the Matthiessen rule. In this work, we consider the Umpklapp (U) scattering, grain boundary scattering(GB), point defect (PD) scattering, and nanoparticle (NP) scattering as the primary contributors to the total phonon scattering relaxation time, and therefore it can be simplified as[4,5]:

$$\tau^{-1} = \tau_{PD}{}^{-1} + \tau_U{}^{-1} + \tau_{NP}{}^{-1} + \tau_{GB}{}^{-1} = A\omega^4 + B\frac{\omega^2 T}{\nu^3} + C\frac{\omega^4}{\nu^3} + D\frac{\nu}{L} (S13)$$

Where $\tau_{PD}{}^{-1}$,$\tau_U{}^{-1}$, $\tau_{NP}{}^{-1}$ $and$ $\tau_{GB}{}^{-1}$ are the phonon relaxation times for PD, U, NP, and GB scattering, respectively, and L is the grain size obtained from the Rietveld refinement (**Table S3, S4, Fig S7**).

The Deybe-Callaway fitting parameters were used to ascertain the influence of the differing scattering modes as described by Li *et al.*[4]. The order of magnitude of the fitting parameters obtained for pristine MgAgSb in said paper were used as initial fitting parameters for our model. The fitting parameters were then iteratively refined using least squares fitting. The necessary data to implement the model and the fitted values for A, B, C, and D can be found in **Table S2.**

## Supplementary Tables

***Table S1***: *Hall mobility and carrier concentration for the coated and uncoated samples.*

| ***Sample*** | $n_H$ ***($10^{19}$ cm$^{-3}$)*** | $\mu_H$ ***(cm$^2$V$^{-1}$s$^{-1}$)*** |
|---|---|---|
| Pristine $\alpha$-MgAgSb | 1.84 | 82.4 |
| MgAgSb-10 cycles $Sb_2Te_3$ | 2.16 | 73.0 |
| MgAgSb-20 cycles $Sb_2Te_3$ | 1.92 | 67.5 |
| MgAgSb-30 cycles $Sb_2Te_3$ | 2.22 | 61.3 |
| MgAgSb-50 cycles $Sb_2Te_3$ | 1.95 | 76.4 |

***Table S2***: *Experimental data and fitted parameters obtained for the Debye-Callaway Model*

| ***Sample*** | ***A ($10^{-40}$ s$^3$)*** | ***B ($10^{-8}$ m$^3$K$^{-1}$s$^{-2}$)*** | ***C ($10^{-30}$ m$^3$s$^{-3}$)*** | ***D ($10^{-6}$)*** |
|---|---|---|---|---|
| Pristine $\alpha$-MgAgSb | 3.8767 | 1.6630 | 3.4715 | 5.8185 |
| MgAgSb-10 cycles $Sb_2Te_3$ | 4.0120 | 1.7114 | 3.5530 | 5.5685 |
| MgAgSb-20 cycles $Sb_2Te_3$ | 6.3651 | 1.001 | 5.0355 | 64.48839 |
| MgAgSb-30 cycles $Sb_2Te_3$ | 3.7861 | 1.6081 | 3.4810 | 9.3029 |
| MgAgSb-50 cycles $Sb_2Te_3$ | 3.7275 | 1.488 | 3.3839 | 5.9125 |

***Table S3***: *Refined lattice parameters and crystallite size*

| ***Structure model*** | ***Space group*** | ***a (Å)*** | ***b (Å)*** | ***c (Å)*** | ***γ (°)*** | ***V (Å$^3$)*** | ***Phase content (wt%)*** | ***L (nm)*** |
|---|---|---|---|---|---|---|---|---|
| $\alpha$-MgAgSb | *I*-4*c*2 | 9.1743(2) | 9.1743(2) | 12.7157(3) | - | 1070.25(7) | 91 | 150 |
| $Ag_3Sb$ | *Pmm*2 | 2.9959(4) | 4.8520(5) | 5.2517(6) | - | 76.34(3) | 6 | 35 |
| Sb | *R*-3*m* | 4.3103(4) | | 11.331(3) | 120 | 182.32(8) | 3 | 50 |

***Table S4***: *Longitudinal, shear, and average velocity of sound and Debye temperature calculated from it*

| ***Sample*** | $v_L$***(ms$^{-1}$)*** | $v_S$***(ms$^{-1}$)*** | $v$***(ms$^{-1}$)*** | $\theta_D$ ***(K)*** |
|---|---|---|---|---|
| Pristine $\alpha$-MgAgSb | 3532.2 | 1656.2 | 1864.4 | 197 |
| MgAgSb-10 cycles $Sb_2Te_3$ | 3550.3 | 1660.3 | 1869.2 | 198 |
| MgAgSb-20 cycles $Sb_2Te_3$ | 3498.1 | 1624.4 | 1829.4 | 193 |
| MgAgSb-30 cycles $Sb_2Te_3$ | 3466.8 | 1614.2 | 1817.7 | 192 |
| MgAgSb-50 cycles $Sb_2Te_3$ | 3547.5 | 1650.4 | 1858.6 | 197 |

**Supplementary Figures**

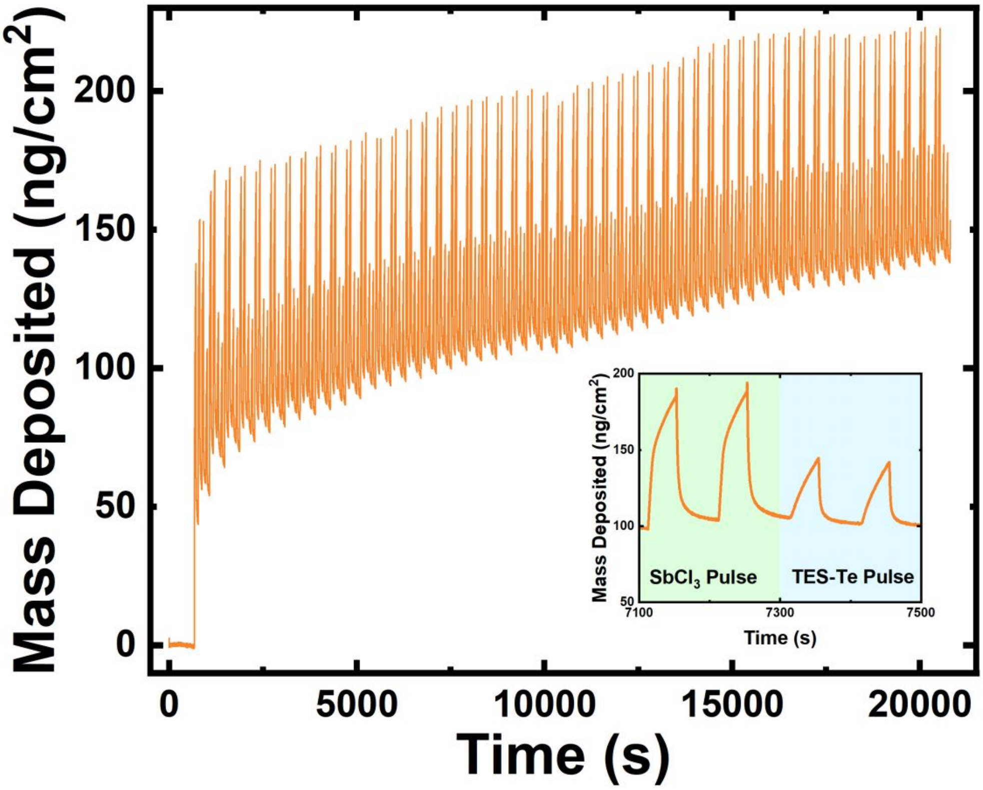


***Figure S1**: QCM data of the ALD process for super-cycling $SbCl_3$ and TES-Te.*

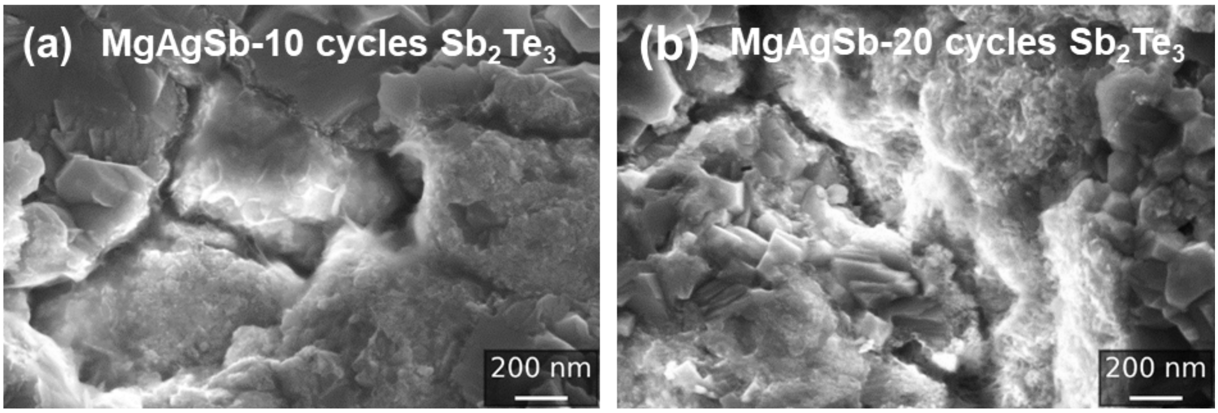


***Figure S2**: SEM fractogram of a) pristine α-MgAgSb and (b) MgAgSb-20 cycles $Sb_2Te_3$.*

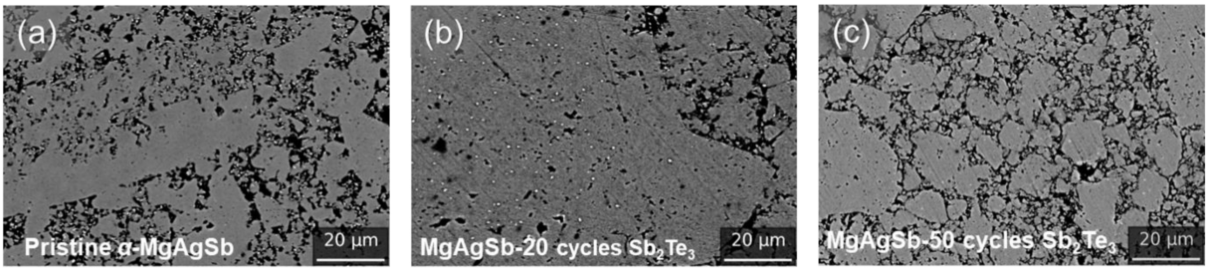


***Figure S3***: *SEM BSD images of (a) Pristine α-MgAgSb, (b) MgAgSb-20 cycles* $Sb_2Te_3$*, and (c) MgAgSb-50 cycles* $Sb_2Te_3$*.*

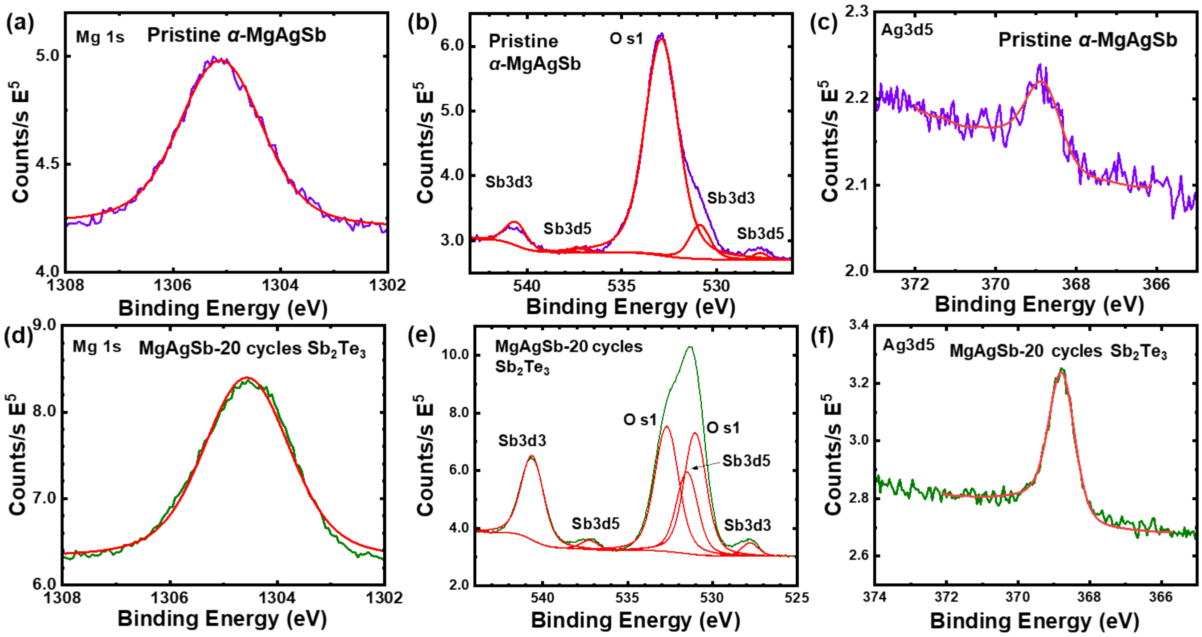


***Figure S4***: *(a-c) High resolution XPS spectra of Mg 1s, Sb* $3d_{3/2}$ *and Sb* $3d_{5/2}$*, and Ag* $3d_{5/2}$ *for pristine α-MgAgSb and (d-f) MgAgSb-20 cycles* $Sb_2Te_3$*.*

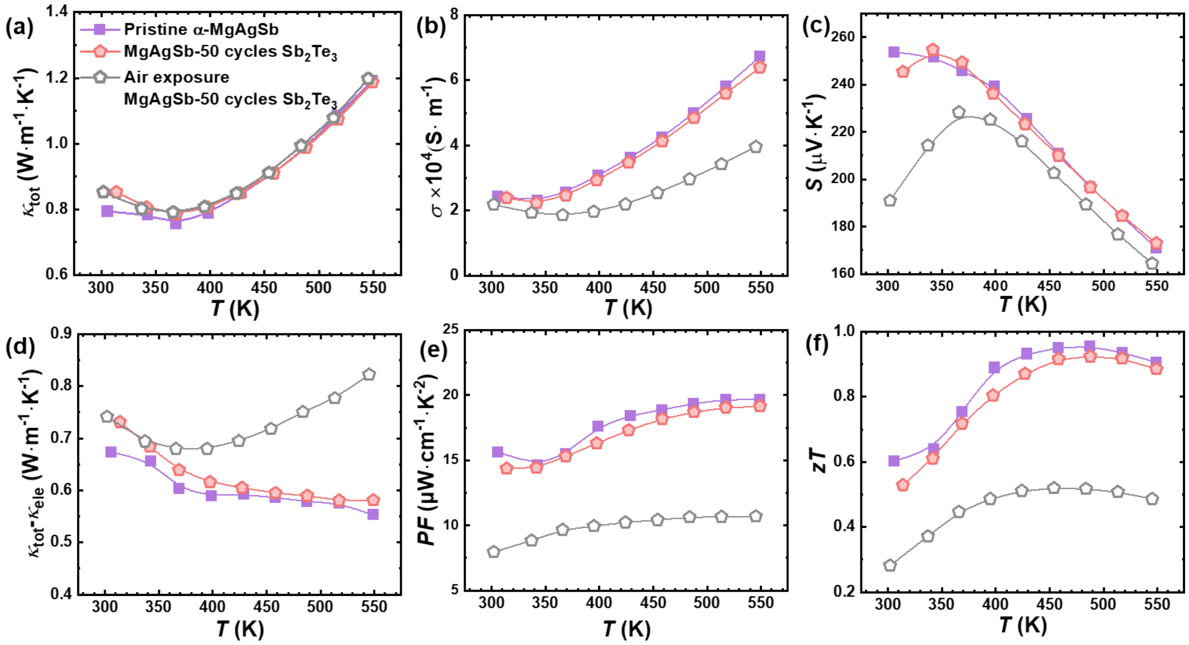


***Figure S5***: *Temperature dependence of (a) total thermal conductivity, (b) electrical conductivity, (c) Seebeck coefficient, (d) total thermal conductivity minus the electronic contribution, (e) Power Factor, (f) Figure of merit zT for the samples processed outside (Air) and inside (Argon) a glovebox.*

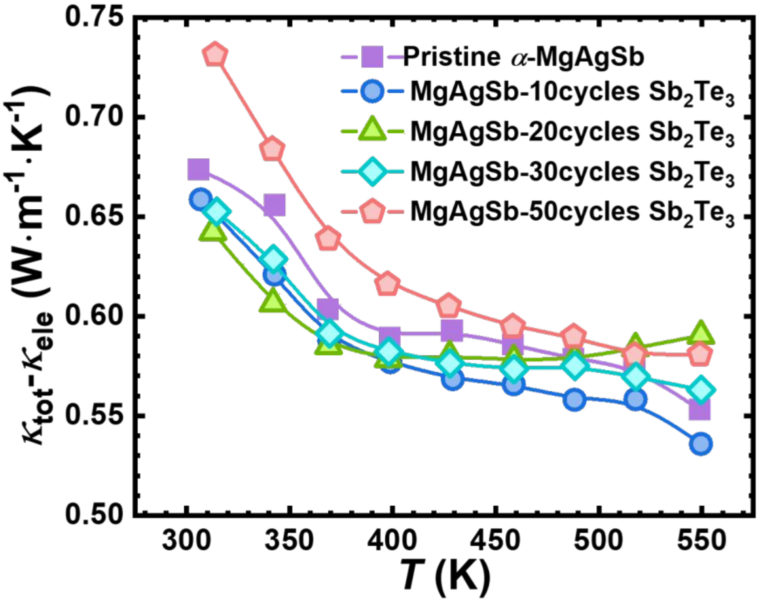


***Figure S6***: *Temperature dependence of the lattice thermal conductivity for coated and uncoated α-MgAgSb samples*

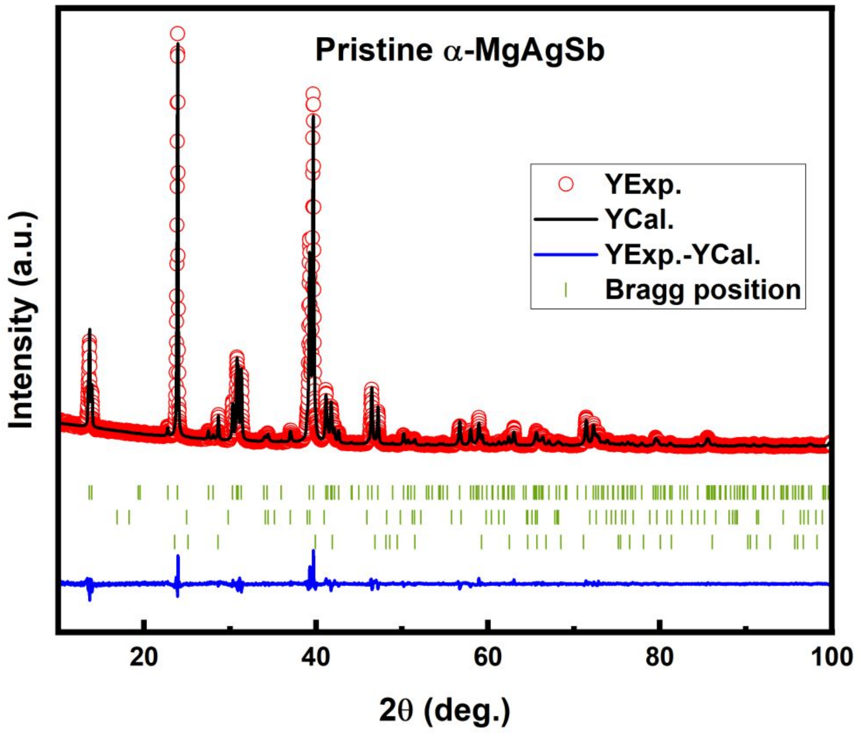


***Figure S7**: Experimental (red, dotted line) and calculated diffraction (black, solid line) patterns and the difference profile (blue solid line near the 2θ scale) and the Bragg positions of the different phases (green, vertical bars) of pristine α-MgAgSb*

## References


(1) Zhu, J.; Zhang, X.; Guo, M.; Li, J.; Hu, J.; Cai, S.; Cai, W.; Zhang, Y.; Sui, J. Restructured Single Parabolic Band Model for Quick Analysis in Thermoelectricity. *npj Comput. Mater. 2021 71* **2021**, *7* (1), 1–8. https://doi.org/10.1038/s41524-021-00587-5.

(2) Kurosaki, K.; Kosuga, A.; Muta, H.; Uno, M.; Yamanaka, S. Ag9TlTe5: A High-Performance Thermoelectric Bulk Material with Extremely Low Thermal Conductivity. *Appl. Phys. Lett.* **2005**, *87* (6), 61919. https://doi.org/10.1063/1.2009828/331068.

(3) Sanditov, D. S.; Belomestnykh, V. N. Relation between the Parameters of the Elasticity Theory and Averaged Bulk Modulus of Solids. *Tech. Phys.* **2011**, *56* (11), 1619–1623. https://doi.org/10.1134/S106378421111020X/METRICS.

(4) Li, A.; Wang, L.; Li, J.; Mori, T. Global Softening to Manipulate Sound Velocity for Reliable High-Performance MgAgSb Thermoelectrics. *Energy Environ. Sci.* **2024**, *17* (22), 8810–8819. https://doi.org/10.1039/D4EE03521F.

(5) Callaway, J.; Von Baeyer, H. C. Effect of Point Imperfections on Lattice Thermal Conductivity. *Phys. Rev.* **1960**, *120* (4). https://doi.org/10.1103/PhysRev.120.1149.
